# Ge-Photodetectors for Si-Based Optoelectronic Integration

**DOI:** 10.3390/s110100696

**Published:** 2011-01-12

**Authors:** Jian Wang, Sungjoo Lee

**Affiliations:** Department of Electrical and Computer Engineering, National University of Singapore, 4 Engineering Drive 3, 117576 Singapore, Singapore; E-Mail: wangjian@nus.edu.sg

**Keywords:** germanium, photodetector, Si photonics

## Abstract

High speed photodetectors are a key building block, which allow a large wavelength range of detection from 850 nm to telecommunication standards at optical fiber band passes of 1.3–1.55 μm. Such devices are key components in several applications such as local area networks, board to board, chip to chip and intrachip interconnects. Recent technological achievements in growth of high quality SiGe/Ge films on Si wafers have opened up the possibility of low cost Ge-based photodetectors for near infrared communication bands and high resolution spectral imaging with high quantum efficiencies. In this review article, the recent progress in the development and integration of Ge-photodetectors on Si-based photonics will be comprehensively reviewed, along with remaining technological issues to be overcome and future research trends.

## 1. Introduction

In the past decade, Si photonics has become one of the hottest research domains in the World since it holds great promise for maintaining the performance roadmap known as Moore’s Law. As short-distance data exchange rates approach 10 Gb/s, metal interconnection is facing a number of inevitable issues such as slow resistance-capacitance limit speed and large heat dissipation. Under these circumstances, it is well known that for data communication beyond 10 Gb/s, optical signal delivery is more advantageous compared to today’s copper interconnections. As a result, combining sophisticated process techniques, low cost and mass production, Si based Electro-Photonic Integrated Circuits (EPIC) emerge as one of the most promising solutions for next generation interconnection techniques. In fact, long-haul combinations have been based on fiber optics techniques for the last 30 years. The wavelength used for the majority of long-distance data transitions is in the 1.3–1.55 μm range, corresponding to the minimum loss window of silica optical fiber. If the same wavelength can be utilized in the future short-distance data transfers including inter-chip, chip-to-chip and Fiber-To-The-Home (FTTH) communications, all end users will be able to connect directly to the external servers without the need for wavelength conversion, making global communication much easier and cheaper. As a result, Si EPIC working in 1.3–1.55 μm wavelength has become aggressively pursued by researchers worldwide.

To date, enormous efforts have been invested in Si photonics techniques and critical breakthroughs and milestones have been achieved. Various passive components [1], active devises like lasers [2], and high speed modulators [3] have been reported. Being the device that ends the optical path, photodetectors, which convert light back into electrical signals, are vital component for Si photonic integrated circuits. In fact, the trigger of the past decade’s Si photonics upsurge was the first successful demonstration of the high-efficiency Germanium photodetector [4]. Although Si photodetectors have been widely used in optical receivers in the wavelength range around 850 nm, its relatively large bandgap of 1.12 eV corresponding to an absorption cutoff wavelength of ∼1.1 μm hinders Si photodetectors’ application in the longer wavelength range of 1.3 and 1.55 μm. For a more seamless integration with current long-haul communication technology, a material with strong absorption coefficient in the 1.30–1.55 μm is very desirable.

Among the available choices, III–V compound semiconductors possess the advantage of high absorption efficiency, high carrier drift velocity and mature design and fabrication technology for optical devices. Therefore, integration of high performance III–V photodetectors onto the Si platform by flip-chip bonding or direct heteroepitaxy has been widely reported. However, the introduction of III–V materials into Si process is at the expense of high cost, increased complexity and potential introduction of doping contaminants into the Si CMOS devices since III–V materials also act as dopants for group IV materials.

Germanium, a group IV material the same as Si, avoids the cross contamination issue. Though Ge is also an indirect bandgap (E_g_ = 0.66 eV) material like Si, its direct bandgap of 0.8 eV is only 140 meV above the dominant indirect bandgap. As a result, Ge offers much higher optical absorption in 1.3–1.55 μm wavelength range, thus making Ge-based photodetectors promising candidates for Si photonics integration. However, the 4% lattice mismatch between Ge and Si places challenging obstacle towards monolithic integration of high-quality low dislocation density Ge devices through Ge on Si heteroepitaxy. Nevertheless, to date, device-grade single-crystalline Ge films have been demonstrated by many groups with practical high performance Ge photodetectors.

In this review paper, we first introduce in Section 2 the various Ge growth techniques. Different photodetector electrical structures and light coupling schemes are briefly described in Sections 3 and 4, respectively. In Section 5, the historical research trends along with the performances of Ge photodetectors reported by various research groups are summarized, along with the remaining technical issues and future research directions. Conclusions are presented in Section 6.

## 2. Ge Growth Techniques

Tracing back in history, the first Ge on Si detector was reported in 1984 by Luryi *et al.* [5]. The demonstrated detector showed 41% quantum efficiency at a wavelength of 1.45 μm, where an MBE-grown 1,800 Ǻ n + Ge_x_Si_1-x_ alloy (graded in ten steps from x = 0 to x = 1) acted as a buffer layer for the heteroepitaxy of Ge on Si. Since then, various techniques with their own pros and cons have been pursued for the growth of Ge films on Si surfaces. The main quality criterion of the Ge layer can be categorized as: procedure complexity, material cost, growth temperature, and the resulting Ge layer’s dislocation density and strain.

### 2.1. Poly Ge Films

**Figure 1.**
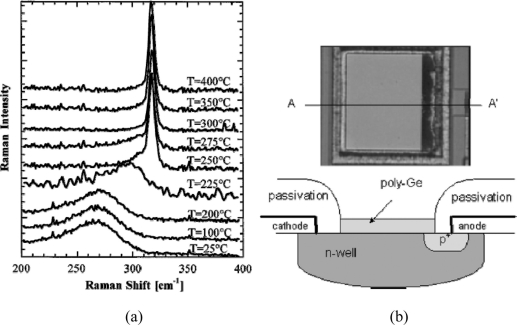
**(a)** Raman spectra of the Ge on Si samples grown at different temperatures by thermal evaporation method. From [6]. **(b)** Photograph of one pixel of the digital camera (top) and a sketch of its cross section. From [8].

For ease of integration of near-infrared detectors with standard Silicon process lines for signal acquisition, amplification and processing, low temperature growth of Ge layers is much desired. In 2000, a Ge deposition approach based on the thermal evaporation with process temperatures as low as ∼300 °C was first proposed in the pioneering work conducted by Masini *et al*. [6]. It was found that polycrystalline Ge deposition can be possible at substrate temperatures as low as 300 °C, as confirmed by the Raman spectra results (Figure 1). This method allows simple and low cost integration with Si processes. Monolithic integration of an array of eight polycrystalline Ge pixels with CMOS readout electronics was demonstrated based on this method [7], shortly after which Colace *et al*. [8] reported the realization of a digital camera, further confirming the process compatibility of the low-temperature approach.

Moreover, although the low temperature deposition introduces a relatively high density of defects and dislocations into the poly-Ge layer and worsens the electrical properties compared to crystalline Ge films, it was shown recently that by a careful design, acceptable performance of the polycrystalline Ge photodetector for Si photonics integration can be obtained, with responsivities between 0.1 A/W and 0.3 A/W [9].

### 2.2. Crystalline Ge Growth with Graded SiGe Buffer Layers

In the early stage of crystalline Ge film epitaxy on Si wafers, a compositionally graded SiGe region was commonly adopted as buffer layer. This approach was first adopted in the SiGe/Si system by Luryi *et al*. [5] and later improved by Fitzgerald *et al*. in 1990 [10]. Multiple buffer layers with increasing Ge content were adopted to relax the high strain between Ge and Si, which minimizes dislocation nucleation and reduces the threading dislocations. The final strain-relaxed Si_1-x_Ge_x_ layers grown on these graded layers showed low density of threading-dislocations, 4 × 10^5^ cm^−2^ for x = 0.23 and 3 × 10^6^ cm^−2^ for x = 0.50.

However, the graded SiGe buffer method usually requires a thick 10 μm buffer for pure Ge epitaxy on Si, while in modern Si photonics technology, Ge photodetectors are favorably fabricated in close adjacency with Si optical waveguide facilitating evanescent or butt-coupling of the optical power. As a result, a new technique with thin buffer layers is still needed.

### 2.3. Two Step LT/HT Ge Growth

The origin of the two-step LT/HT (low temperature/high temperature) growth technique can be traced back to 1986 for GaAs growth on Si by Fan *et al*. [11]. Its application in the epitaxially grown Ge on Si was first proposed and utilized by Colace *et al*. [12] in a ultra high vacuum chemical vapor deposition (UHVCVD) growth reactor in 1998, since when it has attracted wide interest for Ge epitaxial growth. In the two-step Ge growth procedure, first, after thorough cleaning, the substrate is maintained at low temperature (∼300–400 °C), and a thin layer of Ge buffer layer (∼50–100 nm) is grown to prevent strain release through undesirable island growth. Second, the substrate temperature is elevated to ∼550–700 °C and a thick Ge layer with reduced threading dislocation density is grown on top of the low-temperature thin Ge buffer. It should be noted that the two-step Ge method can be adopted not only in UHVCVD systems, but also in growth tools such as reduced-pressure CVD (RPCVD) [13] and molecule beam epitaxy (MBE) [14].

The Ge layers growth by two-step Ge epitaxy typically suffers from a high threading dislocation density (TDD) in the order of 10^8^–10^9^ cm^−2^. Therefore, high temperature annealling is employed by many groups to reduce the TDD to an acceptable level. For example, the research of Luan *et al*. indicates that the TDD in two-step Ge layer can be significantly reduced by cyclic thermal annealing. The optimized annealing condition (900 °C/10 min, 780 °C/10 min, cycle number: 10) can reduce the threading dislocation density to ∼2 × 10^7^ cm^−2^ [15,16]. Ge photodetectors based on this process were successfully demonstrated to have improved performance [15,17]. However, the annealing process increases the thermal budget undesirable for photodetectors’ integration with Si MOSFET. Therefore, a number of experiments have been reported to demonstrate high Ge detector performance which are based on low-temperature anneal or even no additional thermal anneal [18,19]. In Table 1, some of the currently active groups’ Ge growth methods are summarized.

### 2.4. Other Ge Growth Methods

**Figure 2.**
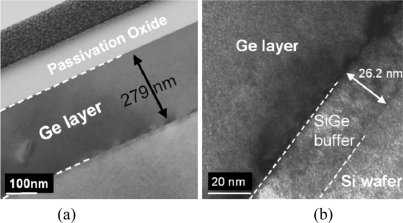
**(a)** HR-TEM image of epitaxial Ge layer using two-step Ge growth method combining with an intermediate SiGe buffer layer. **(b)** Zoom-in image of the heterostructure epitaxial layers of Si/ Si_0.75_Ge_0.25_ /Ge. From [31].

Many attempts to modify the two-step Ge growth procedure have been reported. An UHV-CVD growth of high quality Ge on Si substrate using modified two-step Ge growth method combining with intermediate thin SiGe buffer layers was proposed first by Huang *et al*. in 2004 [26].The buffer region consisted of 0.6-μm-thick Si_0.45_Ge_0.55_ and 0.4-μm-thick Si_0.35_Ge_0.65_ layers. *In-situ* annealling for 15 min at 750 °C was carried out to further reduce the dislocation density. The thickness of the SiGe buffer was further reduced by Nakatsuru *et al*. [27] by employing a 13 nm-thick Si_0.5_Ge_0.5_ buffer layer grown at 450–520 °C. After post-deposition annealling of 800 °C/15 min, the Ge layer shows a low roughness of 0.44 nm. Loh *et al*. [28] also reported an epi-Ge layer based on the SiGe buffer method, where the SiGe buffer is grown at low temperature of 350–400 °C with the thickness of around 30 nm (Figures 2(a,b)).

Another way to improve Ge film quality is H_2_ annealing, which was reported by Choi *et al*. [29]. The demonstrated 800 °C/30 min anneal in H_2_ ambient is able to effectively improve the Ge film quality in terms of surface roughness and TDD. It is proposed that the increased atom mobility caused by hydrogen/Ge bonding is the main mechanism for the improved film surface planarity and defect density.

Recently, a new Ge epitaxy procedure based on low-energy plasma-enhanced chemical vapor deposition (LEPECVD) was demonstrated [30]. Thanks to the high deposition rates and high concentration of atomic H present in the chamber, Ge films with smooth surfaces and TDD ∼2 × 10^7^ are achieved under low thermal budget. Moreover, the fabricated diode shows much lower dark current compared to the devices from UHVCVD method with comparable dislocation density. This is attributed to the improved passivation resulting from the dense plasma in the LEPECVD which is known to be efficient in generating atomic hydrogen radicals.

## 3. Photodetector Electrical Structures

Until now, a number of Ge based photodetectors with different structures are reported. Brief descriptions of typical photodetector structures will be given here for better understanding.

### 3.1. PIN Detectors

PN junctions are one of the most commonly used configurations for semiconductor photodetectors. The PIN diode where “I” stands for intrinsic, includes an intrinsic region in between the P and N regions. Due to the built-in potential or external reverse bias, the intrinsic region is depleted and has high resistivity, so that the voltage drop takes place mainly in this region, giving rise to high electric fields for effective collection of photo-generated electron-hole pairs (EHP). In this configuration, the thickness of the intrinsic region is always many times larger than the highly-doped regions so that most of the EHP’s are generated within the intrinsic region where the strong electric field helps to sweep the EHP to the adjacent p+/n+ region faster than diffusion. Another advantage of the PIN structure is that the depletion-region thickness (the intrinsic layer) can be tailored to optimize both the quantum efficiency and response bandwidth. In Ge PIN photodetectors, while the photoabsorption intrinsic layer is usually Ge for effective absorption around 1.55 μm, the p+ and n+ region can be formed either by implantation [32] or *in-situ* doping to form p+ and n+ regions for the PIN structure [14]. Another way is to use p+/n+ single crystalline Si substrates or deposited polycrystalline Si heterojunctions [33].

### 3.2. Metal-Semiconductor-Metal (MSM) Detectors

PIN photodiodes produce a voltage drop across the diode terminals in response to an external optical input. Such devices are categorized as photovoltaic devices. On the other hand, MSM photodetectors are photoconductive devices whose conductivity is altered when an optical illumination is imposed. Therefore, MSM photodetectors are only functional under non-zero external bias. MSM photodetectors possess the advantage of low capacitance and relative ease of fabrication. The intrinsically low capacitance resulting from its configuration has always been utilized to fabricate high-speed large area detectors. One issue in early Ge MSM photodetectors was their high dark current density, which gives rise to high stand-by power consumption, thus making Ge MSM photodetectors unfavorable and impractical. Due to the narrow bandgap and strong Fermi-level pinning of the metal/Ge interface at valence band, hole injection over Schottky Barrier Height (SBH) is the major component of the dark current in Ge MSM detectors. Regarding this issue, application of dopant segregation (DS) to Ge MSM photodetectors for dark current suppression was experimentally demonstrated by Zang *et al.* [34–36]. Metal-Ge Schottky barrier height modification by an intermediate layer of large bandgap material such as amorphous Ge and SiC is also proposed [37]. While the demonstrated Ge MSM detectors are able to achieve dark current suppression of two to four orders of magnitude, it is still an open question whether these MSM Ge photodetectors are competitive with PIN devices.

### 3.3. Avalanche PD

The simplest avalanche photodiode (APD) has a similar device structure to a p-i-n photodiode. However, a voltage close to its breakdown is usually applied to APD for detection of low power signal with high sensitivity. Under sufficiently higher external bias, electrical field in the photodiode’s depletion region becomes high enough to initiate impact ionization which is responsible for carrier multiplication. Therefore, one absorbed incoming photon does not only generate one electron/hole pair but rather a large number of EPHs leading to a quantum efficiency potentially large than unity. The most important performance indices for APD is excess noise factor quantified by effective ratio of electron and hole ionization rate (k_eff_), gain-efficiency product and sensitivity.

### 3.4. Dark Current Criteria for Photodetectors

An important issue in integrated photodetectors is dark current, which increases the power consumption of the receiver. Most importantly, shot noise associated with this leakage current undesirably degrades the Signal-to-Noise Ratio (SNR) leading to increased bit error rates (BERs).

Generally, dark currents less than 1 μA are referred to as acceptable value for a high-speed receiver design, below which the transimpedance amplifier (TIA) noise is the main noise source [23,38,39]. In practice, a precise value of the required dark current depends upon the speed of operation and the amplifier design. In the recent successful demonstration of an Ge-on-Si photodetector-based receiver, photodetectors with dark current of both ∼10 nA [38] and ∼2 μA [24] were reported. Depending on the receiver design, a higher dark current level is tolerable with certain sacrifices in the receiver parameters. For example, Vivien *et al*. [40] have shown that with an increase of the input power of about 20% in comparison with photodetector without dark current, a photodetector with 300 μA dark current is still able to ensure a BER of 10^−18^ at a frequency close to 50 GHz. The conclusion was drawn based on SPICE simulation taking into account of feedback resistance noise, the shot noise from detector dark current and photocurrent sources, and the transistor channel noise [41]. For the detailed modeling of the precise criteria for the dark current in high speed receiver, the readers are referred to [42] for further understanding.

## 4. Ge Photodetector Light Coupling Schemes

### 4.1. Normal Incidence Photodetectors and the Bandwidth-Efficiency Tradeoff

Normal incidence (NI) photodetectors are also known as vertical photodetectors or surface illuminated photodetectors. Normal incidence is the simplest light coupling scheme with incoming light illuminated on the top or bottom surface of the detector. Almost all the electrical structures, *i.e.*, PIN, MSM and avalanche, can be fabricated in the fashion of NI photodetectors.

Due to its low process complexity, NI photodetectors are widely used in communication technologies. However, they suffer from an inherent drawback due to the bandwidth-efficiency tradeoff. This tradeoff results from the opposite requirement of the thickness of the photoabsorption layer for high bandwidth and high efficiency [43]. The carrier-transit-time-limiting bandwidth *f_t_* can be expressed as [44]:

(1) $$f_{t}≅0.45\times \frac{\upsilon }{d}$$

**Figure 3.**
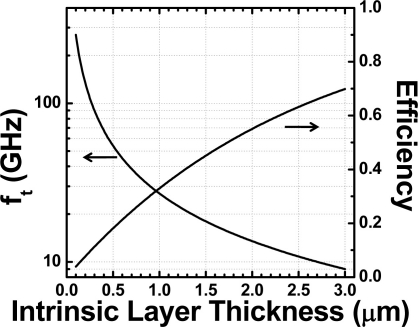
A Calculated carrier-transit-time-limiting bandwidth and efficiencies of normal incidence PIN Ge photodetector.

While the ideal efficiency *η* assuming zero reflection and full carrier collection is:

(2) $$\eta ≅1-e^{-\alpha \times d}$$

where *υ* is carrier transit velocity, d is intrinsic region’s thickness and α is material’s absorption coefficient. Using υ = 6 × 10^6^ cm/s for Ge and α = 4,000 cm^−1^, the carrier-transit-time-limiting bandwidth and efficiencies *versus* intrinsic region thickness can be plotted as Figure 3. As can be seen, for a Ge device with 3dB bandwidth of 100 GHz, an intrinsic layer thinner than 0.27 μm is required with a resulting efficiency of ∼10%.

### 4.2. Resonant Cavity Enhanced (RCE) Detectors

To overcome the tradeoff between bandwidth and efficiency in NI detectors, one method is to sandwich a thin layer of photo absorbing material between two light reflectors so that cavity resonance is enhanced [45,46]. In this structure, light is ideally trapped between the two reflectors and travels through the center light absorber multiple times until fully absorbed. At the same time, the photoabsorption layer can be thin enough to achieve high bandwidth. Another advantage of RCE detectors is the wavelength selectivity. When the light reflector is fabricated in the form of a Bragg reflector, only light in a small range of certain wavelengths is reflected effectively so as to produce high efficiency. The RCE device’s light selectivity makes it especially useful for wavelength division multiplexing (WDM) systems.

**Figure 4.**
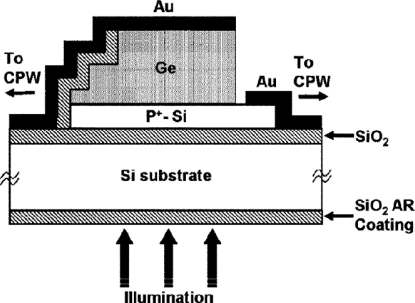
Cross-sectional view of the back-illuminated Ge-SOI Schottky photodetector. From [45].

Ge RCE Schottky photodetectors (Figure 4) were demonstrated by Dosunmu *et al*. [45] in 2005. The resonant cavity was formed between the Au reflecting top metal contact and the SOI substrate. The backside of the SOI wafer was polished to facilitate light coupling. Schottky contact was formed between the top contact Au and the Ge layer while the bottom contact of Au and p+-Si was ohmic contact. The resonant wavelength was found at around 1,538 nm, leading to an increased quantum efficiency of 59%.

Although RCE photodetectors solve the bandwidth-efficiency tradeoff to some extent, the fabrication of high reflectivity mirrors increase the design and process complexity significantly. The multiple layers needed for effective reflection also make RCE detectors difficult integrate with other functional devices. Therefore, other methods with more process and integration friendliness are still required.

### 4.3. Waveguide Photodetectors

**Figure 5.**
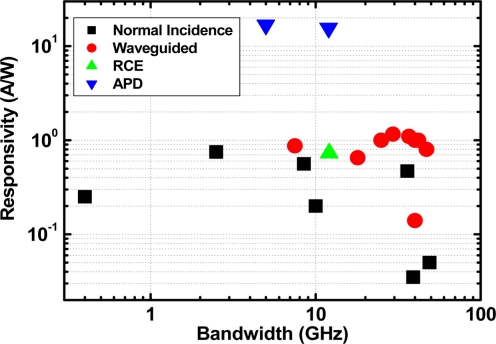
Bandwidth and responsivity of selected Ge photodetectors.

Waveguide integrated photodetectors have been considered to be one of the most promising candidates for overcoming the bandwidth-efficiency tradeoff in normal incidence detectors. In this configuration, a light signal is delivered to the device by in-plane optical waveguide rather than top down, permitting the bandwidth and efficiency to be determined almost independently because the efficiency is specified no longer by the photoabsorption layer thickness, but rather by the waveguide length. Furthermore, large scale integration of Si optical and electrical devices requires all devices to be fabricated on the same planar wafer, which makes an optical waveguide indispensable. Thus integration of a waveguide with photodetectors seems to be a natural choice. The development of Ge-on-Si photodetectors has been going on for more than ten years. In Table 2 and Figure 5, performances reported for some typical Ge photodetectors are summarized.

## 5. Research Trends in Ge Photodetectors

In this section, historical research trends are identified and described. The remaining technical issues and future directions are also discussed here.

### 5.1. Normal Incidence to Waveguide Integration

At the starting stage of Ge-on-Si photodetector development for Si photonics applications, normal incidence Ge photodetectors were first fabricated and comprehensively studied [4,14,17,18,22,33,50,53,56–64] due to their ease of processing.

**Figure 6.**
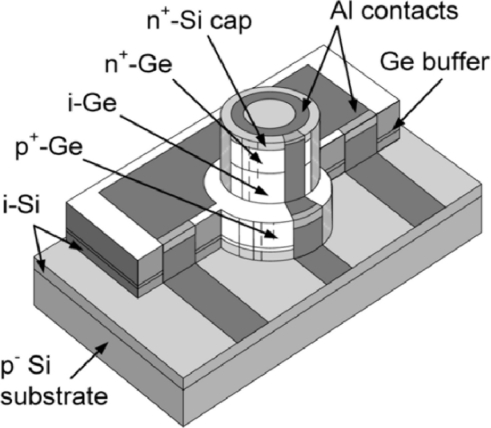
3D view of the PIN Ge photodiode with 49 GHz bandwidth. From [50].

Due to the bandwidth-efficiency tradeoff, typical NI incidence Ge photodetectors offer moderate quantum efficiencies and bandwidths. Among the reported NI photodetectors, Klinger *et al*. [50] reported the highest bandwidth of 49 GHz for Ge-based photodetectors. The Ge p-i-n photodiode was fabricated in Ge grown by MBE two-step Ge growth (Figure 6). Given the nominal Ge intrinsic layer thickness of 300 nm, detector diameter of 10 μm and the series resistance of 25 Ω, the theoretical bandwidth of 54.3 GHz corresponds well with the experimental value. It should be noted that the reported improved 3 dB frequency of 49 GHz from previous result (39 GHz) [14] is mainly due to the reduced series resistance (Rs) of 15 Ω from 32 Ω. The reported responsivity at 1,550 nm is ∼0.05 A/W limited by small device footprint and relatively large density of defects in the Ge layer.

In terms of high responsivity, a thick Ge absorption layer is need. The highest reported value at 1,550 nm wavelength for NI photodetector is 0.75 A/W from a PIN diode with ∼4 μm thick Ge layer fabricated and reported by Fama and coworkers [17]. The Ge layer was epitaxially grown on a Si substrate by two-step UHVCVD combined with high temperature cyclic annealling for the reduction of dislocation density. A time response of less than 200 ps and operation >2.5 Gb/s was also demonstrated.

The study of the Ge normal incidence photodetectors provides valuable insights into the Ge/Si system and its properties. As the Ge growth technique becomes mature and the particulars of Ge/Si devices have been studied in detail, research has gradually been redirected to the integration of Ge photodetectors on Si waveguides to decouple the tradeoff between bandwidth and efficiency mentioned above. Since Si photonics also require devices to be monolithically integrated on the same Si substrate using an on-wafer optical waveguide, integration of Ge photodetectors with waveguides seems mandatory.

To date, a number of groups have demonstrated waveguided Ge photodetectors, including MIT [1,23,65–67], IEF [20,40], IME [35,48,54,68,69], INTEL [47,70], IBM [52,55,71] and Kotura [19,72]. Both PIN and MSM structures are reported in these waveguide photodetectors with comparable performance and high speeds of around 40 GBit/s.

### 5.2. Improvement of Speed Performance of Waveguide Ge Photodetectors

Another trend of the continuous evolvement of Ge photodetectors is the increase of detector bandwidth. At the starting point of Ge detectors’ integration with Si waveguides, Ge growth on SOI wafers and optical coupling between Ge detectors and Si optical waveguides was first explored. The reported detectors are ∼100 μm long to ensure full absorption of light around 1.55 μm wavelength, inevitably leading to large device capacitance so that the bandwidth are limited <10 GHz by RC delay.

Nowadays, special care in design is given to detector’s bandwidth performance. With adoption of shorter devices, sophisticated radio-frequency coplanar waveguides (CPW), metal interconnections and frequency measurement technologies, 40 Gbit/s operation was reported by several groups [47,49,52,73], with waveguide detector bandwidths as high as 47 GHz [73].

### 5.3. Zero-Bias PIN Photodiode

As discussed in Section 3.4, high dark current leads to high stand-by power consumption in addition to the degraded SNR. Moreover, it is desirable for the detector and the receiver circuit to operate on a single power supply which often restricts the bias voltage for photodetector to be less than 1.5 V [74]. As a result, there has been an increasing research interest in the development of low-bias or even zero bias PIN photodiodes.

**Figure 7.**
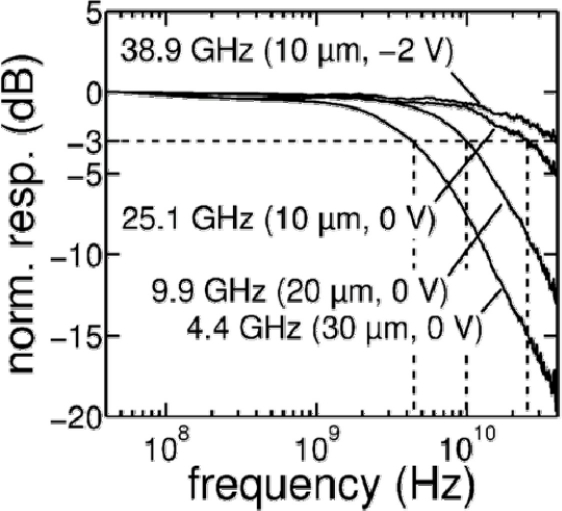
Normalized frequency response at a wavelength of 1,552 nm for PIN detectors with diameters varying from 10 to 30 μm. From [14].

In terms of responsivity, Liu *et al*. [33] measured their Ge PIN detector’s responsivity over the wide spectrum of 650 nm–1,650 nm. The reported responsivity at 0 V bias is more than 98% of the saturated value at 2 V reverse bias, which was attributed to high carrier collection efficiency resulting from the high built-in electric field in the diode’s depletion region. High speed operation at zero-bias was demonstrated by Jutzi *et al.* [14]. From PIN Ge detectors with diameter of 10 μm, a record high zero-bias 3 dB bandwidth of 25.1 GHz was obtained (see Figure 7).

### 5.4. Avalanche Photodetectors

For Ge photodetectors’ application in Si photonics IC, a next level pre-amplifier is necessary to further transform the current signal into a voltage signal for later CMOS IC processing. Avalanche photodetectors offers much lower signal-to-noise ratio compared to PIN or MSM structures. Therefore, more and more interest is being focused on Ge-based avalanche photodetectors. Recently, Intel [53], IBM [55] and IME [54] have all reported successful fabrication of such devices.

**Figure 8.**
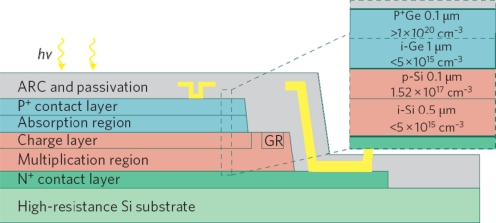
Schematic of Ge/Si APD with a typical SACM configuration. From [53].

The first Ge-based APD was demonstrated by Kang *et al*. [53]. For the reported device, a separate-absorption-charge-multiplication (SACM) configuration (Figure 8) is used to take advantage of both Si’s low noise property and Ge’s strong absorption near 1.55 μm wavelength. The device exhibits low excess noise with low k_eff_ of ∼0.09. The reported sensitivity of −28 dBm at 10 Gb/s is equivalent to a commercial III–V APD and the bandwidth-efficiency product of 340 GHz is even higher than its III–V counterpart, thanks to much lower k value of Si compare to InP material.

Another Ge APD configuration is a conventional MSM structure with nano-engineered metal-to-metal spacing as small as 200 nn reported by Assefa *et al*. [55]. With a low bias voltage of ∼1.5 V, the electric field in the immediate vicinity of the metal contact is already high enough to initiate avalanche amplification. Although the whole APD structure is built on Ge, whose properties are not optimized for APD, the device exhibits a excess noise factor with k_eff_ ∼ 0.2, high speed of ∼40 GHz, and bandwidth-efficiency product of 350 GHz at the wavelength of 1.3 μm. Although the high dark current due to the small metal spacing requires more optimization, the Ge MSM APD shows great potential for the application of Ge in avalanche photodetection.

The APD devices reported above are working at 1.3 μm due to the incorporation of Si into Ge which gives rise to undesired reduction of the absorption efficiency at 1,550 nm. Using two-step Ge growth with a SiGe buffer layer method, the first waveguide-base Ge APD working at 1.55 μm was reported by Ang *et al*. [54]. The device is fabricated based on a SACM structure. A waveguide was used to increase device efficiency and facilitate future Si photonics integration. The reported high responsivity at unity gain was as high as ∼0.8 A/W and a product bandwidth-efficiency of 105 GHz was achieved.

### 5.5. Remaining Technical Issues and Future Research Trends

#### 5.5.1. Pursuit of Higher Bandwidth

**Figure 9.**
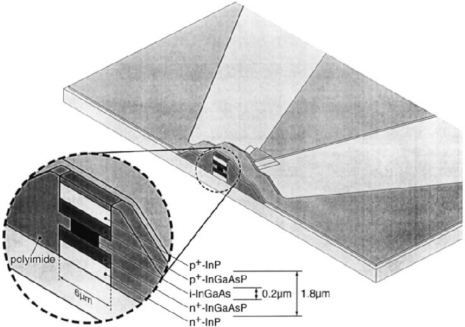
Schematic view of III-V photodetector based on mushroom structure. From [43].

Nowadays, the reported Ge photodetectors’ bandwidths are approaching 50 GHz, ready for near-future 40 Gb/s applications. On the other hand, in correspondence with III–V photodetectors, it can be seen that there is still much room for enhancement. For bandwidths beyond 100 GHz, much thinner Ge intrinsic layers should be used. As in the high frequency region, undesirable parasitic effect such as contact resistance, stray capacitance and inductance may become the main limiting factors in bandwidth performance. Given the fact that reducing the intrinsic region’s thickness for smaller carrier transition time at the same time leads to increase of device capacitance, the mushroom-mesa structure [43] (Figure 9) may be of help for further bandwidth evolution, since it is capable of reducing the Rs and capacitance simultaneously.

#### 5.5.2. Monolithic Integration of Ge Photodetectors with CMOS Integrated Circuits

Essential for future Si EPIC is the co-integration of Ge photodetectors with functional CMOS circuits, which brings optical detection and further signal processing together. Therefore, there has been much effort in pursuing such integration. However, fabrication of high performance Ge photodetectors together with conventional CMOS devices comes with several technical issues that must be addressed, including the thermal budget issue, the cross contamination issues and the non-planarity issue due to Ge layer thickness.

To avoid the high temperatures needed during two-step Ge growth and subsequent cyclic annealling for dislocation density reduction, thermal evaporation polycrystalline Ge with full compatibility with CMOS fabrication due to its deposition temperature as low as ∼300 °C was demonstrated [6]. However, the relatively inferior electrical quality of the Ge limits the performance of the photodetector making this technique not suitable for high-speed high efficiency Si EPIC for future data transmission.

**Figure 10.**
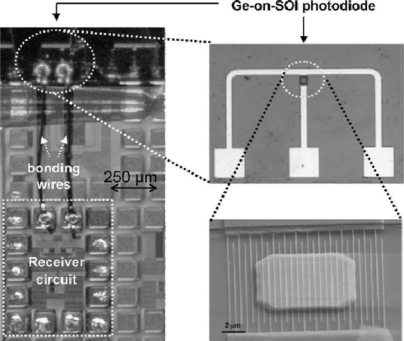
Optical micrograph showing a Ge-on-SOI photodiode wire-bonded to a high-gain CMOS IC. High-magnification optical and SEM micrographs of the photodiode are also shown. From [38].

Another study [38] uses a wire-bonding technique to connect separately-manufactured Ge photodetectors and a Si CMOS amplifier (see Figure 10). While this approach reduces the process complexity and cost considerably, undesirable large parasitic capacitance from the bonding pad and inductance from the bonding wire inevitably sacrifice the speed performance.

**Figure 11.**
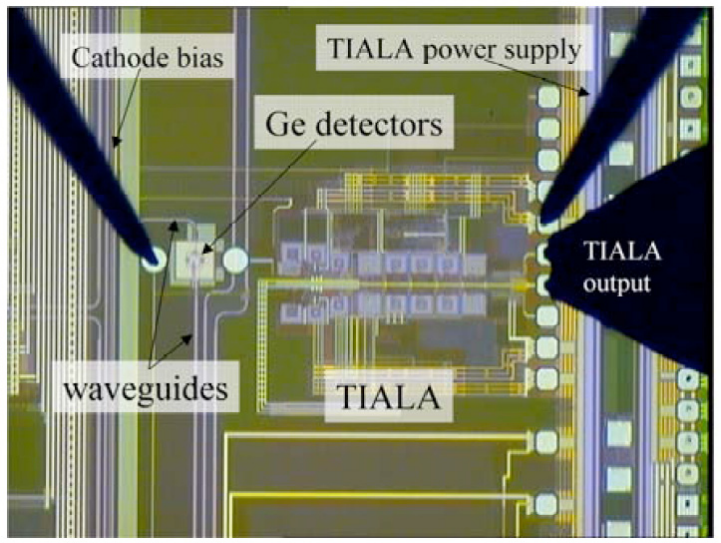
A four-channel, 10 Gbps monolithic optical receiver in 130 nm CMOS with integrated Ge waveguide photodetectors. From [24].

Masini *et al*. [24] reported the first successful monolithic integration of a Ge photodetector with CMOS on the same wafer (Figure 11). Special attention was given to the thermal budget planning in the process design. Since high temperature is compulsory for gate oxide growth, Ge grow was inserted after the gate processing. Furthermore, to avoid damage by high temperature Ge layer epitaxy, reduced-press chemical vapor deposition (RPCVD) at 350 °C was used. Also, no high temperature annealling was carried out. To address the non-planarity issue normally induced by thick Ge of several microns used for efficient light absorption, a waveguided photodetector with a thin Ge layer of ∼200 nm was used. Since with low temperature Ge growth and no curing annealling, the fabricated 25 μm-long detector demonstrated large dark current of 10 μA @−1 V and relatively low responsivity of ∼0.6 A/W at 1,554 nm.

**Figure 12.**
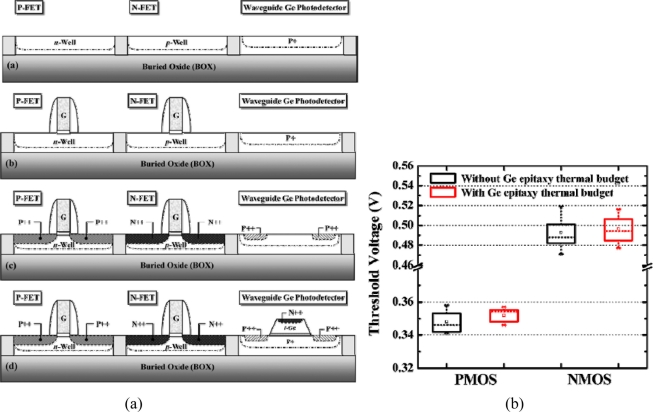
**(a)** Schematic showing the “electronic-first and photonic-last” integration approach for monolithically fabricating the Ge p-i-n photodetector and Si CMOS circuit on common SOI platform. **(b)** Comparable threshold voltages were observed in both n-MOS and p-MOS transistors despite the introduction of an additional thermal budget during the Ge epitaxy growth. From [75].

The impact of high temperature prebake treatment before Ge epitaxy on the monolithic integration was studied by Ang *et al.* [75]. For UHVCVD Ge epitaxy, an 800 °C prebake before actual growth is usually necessary for removal of native oxide from Si surface to ensure Ge epitaxy quality. However, such high temperature causes thermally enhanced dopant diffusion in CMOS devices leading to possible shift in transistor’s threshold voltage (V_th_). This concern is especially the case for short channel devices since the impact of dopant diffusion is larger. It was found that for transistors with effective channel length of ∼180 nm, the additional 800 °C thermal budget virtually have no apparent impact on the V_th_ (Figure 12(b)). However, for the state-of-the-art aggressively scaled down devices with critical dimension well below 100 nm, whether the influence of the 800 °C prebake is still negligible leaves an open question. As a result, research and development of low temperature Ge growth technique with low dislocation density Ge film would be expected to be one of the most focused directions for future research. The reported monolithic integration scheme is also based on “electronic-first and photonic-last” approach (Figure 12(a)) to avoid Ge degradation and cross contamination during the high temperature gate oxidation and dopant activation in Si transistors.

#### 5.5.3. Plasmonics for Extreme Light Concentration

For higher speed, lower noise and suppressed power consumption, photodetectors are being fabricated in smaller dimensions [76]. However, previously the physical dimensions of the photodetectors were limited in the micrometer range by classical diffraction theory.

**Figure 13.**
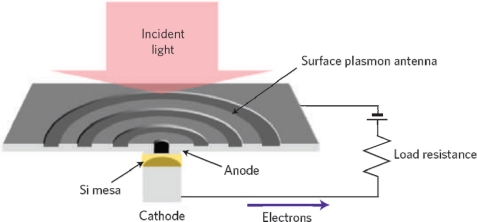
Concentric grating coupler used in the nano-photodiode. From [77].

Recently, the amazing ability of plasmonic structures to concentrate light both laterally and in the depth of a semiconductor material beyond the diffraction limit into the deep-subwavelength-dimension was reported by Ishi *et al.* [77] A concentric grating surface plasmon antenna of 10 μm diameter was demonstrated to concentrate light into the center Si mesa Schottky diode of an active area of 300 nm in diameter (Figure 13). The observed more than 20-fold enhancement in photocurrent confirms the plasmonic effect. The estimated bandwidth of such small detector exceeds 100 GHz.

Because of its promise in Ge photodetector’s drastic miniaturization into the nano-scale domain and expected high speed, plasmonics technology’s application in Ge-based detectors are being actively pursued both theoretically [78] and experimentally [79] making it one of the major research directions in the future.

## 6. Conclusions

This paper summarizes the historical and current trends of Ge-on-Si photodetectors development, which is essential for Si EPIC integration. Various electrical structures (PIN, MSM, and avalanche) and optical coupling schemes (normal incidence, resonant cavity enhancement and waveguide integration) have been adopted in Ge photodetectors, demonstrating high responsivity approaching 100% quantum efficiency and high speed operation at around 40 Gb/s. With practical Si photonics EPIC around the corner, higher speed, easier integration with CMOS fabrication and novel approaches such as plasmonics-enabled nano-scale detector will become the main focuses of research in the near future.

## Figures and Tables

**Table 1.** Summary of recent Ge epitaxy method from selected groups.

	**Group**	**Year**	**Ref.**	**Tool**	**Low Temp. buffer**	**High Temp. Ge**	**Anneal**	**Aneal condition**	**RMS (nm)**	**TDD (cm^−2^)**
**two-step LT/HT growth**	IEF	2004	[13]	RPCVD	400 °C 25 nm Ge	<750 °C 730 nm	yes	750/875 °C, 10 cycles	2.2	<2 × 10^8^
	IEF	2009	[20]	RPCVD	400 °C 40 nm Ge	730 °C 300 nm	yes	not specified	-	-
	Intel	2006	[21]	RPCVD	400 °C 100 nm Ge	670 °C 1.2 μm	yes	900 °C, 15 min	-	∼1 × 10^7^
	IBM	2004	[22]	UHVCVD	350 °C 50 nm Ge	600 °C 400 nm	yes	780/900 °C, 10 cycles	-	∼1 × 10^8^
	Univ. stuttgart	2005	[14]	MBE	thin LT buffer	550° C 1 μm	no	-	-	-
	MIT	1999	[16]	UHVCVD	350 °C 30 nm Ge	600 °C 1 μm	yes	780/900 °C, 10 cycles	-	∼2 × 10^7^
	MIT	2007	[23]	UHVCVD	360 °C 60 nm Ge	730 °C 1.1 μm	yes	650/850 °C, cyclic	-	-
	Luxtera	2007	[24]	RPCVD	no buffer	350 °C 200 nm	no	-	-	-
	Kotura	2010	[19]	CVD	400 °C 100 nm Ge	670 °C 1.1 μm	yes	not specified	-	-
	ETRI	2009	[25]	RPCVD	400 °C 100 nm Ge	650 °C 1.2/1.7 μm	no	-	1.3	-
	Univ. Roma Tre	2006	[18]	UHVCVD	350 °C thin Ge	600 °C 1 μm	no	-	-	-
**SiGe buffer**	Unvi. Texas	2004	[26]	UHVCVD	1 μm SiGe	400 °C 2.5 μm	yes	750 °C, 15 min	-	-
	Canon ANELVA	2006	[27]	UHVCVD	450–520 °C 13 nm SiGe 370 °C 30 nm Ge	550–600 °C 1 μm	yes	800 °C, 15 min	0.44	-
	IME	2007	[28]	UHVCVD	350–400 °C 30 nm SiGe 350–400 °C 30 nm Ge	550–600 °C 100 nm	no	-	1.4	∼1 × 10^7^
**H_2_ anneal**	Stanford	2008	[29]	RPCVD	350 °C 200 nm Ge	600 °C 400 nm	yes	800 °C, 30 min, in H_2_	∼1	0.8–1 × 10^7^
**LEPECVD**	Como	2009	[30]	LEPECVD	no buffer	500–600 °C 1 μm	yes	600/780 °C, 3 cycles	-	2 × 10^7^

**Table 2.** Summary of performances from selected Ge photodetectors.

**Year**	**Reference**	**1st author**	**structure**	**I_dark_ (μA)@−1 V**	**Responsivity @λ = 1.55 µm @−1 V (A/W)**	**Highest 3 dB electrical bandwidth (GHz)**	**APD gain-bandwidth product (GHz)**
2000	[4]	L. Colace	NI PIN	12	0.25	∼0.4@−4 V	-
2002	[17]	S. Fama	NI PIN	1.2	0.75	2.5@−1 V	-
2005	[14]	M. Jutzi	NI PIN	0.08	0.035@0 V	39@−2 V	-
2005	[33]	J. Liu	NI PIN	∼0.8	0.56	8.5@−1 V	-
2005	[45]	O. I. Dosunmu	NI PIN RCE	0.38@−5 V	0.73	12.1@−3 V	-
2006	[18]	L. Colace	NI PIN	∼10	0.2	10@−1 V	-
2007	[40]	L. Vivien	WG MSM	130	1	25@−6 V	-
2007	[23]	D. Ahn	WG PIN	0.9	0.87	7.5@−3 V	-
2007	[47]	T. Yin	WG PIN	0.267@−2 V	1.16@−2 V	29.4@−2 V	-
2008	[48]	J. Wang	WG PIN	0.06	0.65	18@−1 V	-
2009	[49]	L. Chen	WG MSM	4@−5 V	∼1	40@−5 V	-
2009	[50]	S. Klinger	NI PIN	∼0.10	0.05@−2 V	49@−2 V	-
2009	[25],	D. Suh	NI PIN	0.042	0.47	36@−3 V	-
2009	[20]	L. Vivien	WG PIN	∼1	1	42@−4 V	-
2009	[51]	D. Suh	WG PIN	0.072	0.8	47@−3 V	-
2010	[19]	D. Feng	WG PIN	1.3	1.1	36.8@−3 V	-
2010	[52]	S. Assefa	WG MSM	90	0.14	40@−2 V	-
2008	[53]	Y. Kang	NI SACM APD	∼10@25 V	∼15.6@∼25 V@1.3 μm	∼12@∼25 V	340
2010	[54]	K. Ang	WG SACM APD	∼100@23 V	16.8@∼23 V	5@∼23 V	105
2010	[55]	S. Assefa	WG MSM APD	∼100@	-	∼35@1.5 V	350
